# Exploring Employee Perceptions towards Smart Working during the COVID-19 Pandemic: a Comparative Analysis of Two Italian Public Research Organizations

**DOI:** 10.1007/s11115-021-00559-9

**Published:** 2021-09-04

**Authors:** Marco Cellini, Lucio Pisacane, Massimo Crescimbene, Fabio Di Felice

**Affiliations:** 1 CNR-IRPPS: Consiglio Nazionale delle Ricerche, Istituto di Ricerche sulla Popolazione e le Politiche Sociali, Lazio Roma, Italy; 2 INGV: Istituto Nazionale di Geofisica e Vulcanologia, Lazio Roma, Italy

**Keywords:** Smart working, Research institutions, Public institution, COVID-19

## Abstract

Of all the socio-economic changes caused by the Covid-19 pandemic, the disruption to workforce organizations will probably leave the largest indelible mark. The way work will be organized in the future will be closely linked to the experience of working under the same institution’s response to the pandemic. This paper aims to fill the gap in knowledge about smart working (SW) in public organizations, with a focus on the experience of the employees of two Italian research organizations, CNR and INGV. Analysing primary data, it explored and assessed how SW had been experienced following the implementation of governmental measures aimed at limiting the spread of COVID-19.

**Supplementary Information:**

The online version contains supplementary material available at 10.1007/s11115-021-00559-9.

## Introduction

Among the socio-economic changes caused by the Covid-19 pandemic outbreak the disruption to workforce organizations will be the one to most likely leave an indelible mark. The way public and private organizations will organise work in the future will be closely linked to the experiences of the institution’s response to the pandemic. The social distancing and the confinement, imposed to reduce the contagion, forced governments and public organizations to reorganize their workforces into new forms as per pre-existing concepts such as Smart Working (SW), Telework (Huws et al., 1990), Flex Work, Working From Home (WFH), Alternative Work Arrangements (Gil-Garcia et al., 2014). The result has been a large-scale social experiment, both in the public and in the private sectors. For many organizations the governmental responses to the pandemic forced the adoption of SW as the only viable alternative during the emergency, and the experience will give impetus to future schemes as well as an incentive to promote SW.

In the literature there has been a lack of interest in and evidence about the effects of SW adoption, both on the community within which the organisations operate and on employee perception. Previous research has explored the characteristics of teleworking (Alizadeh, 2012) and the relationship between work and family roles including boundaries and gender roles, among home-based teleworkers and their families (Sullivan & Lewis, 2001) along with the issue of digital divide, skills, and perceptions (Petrillo et al., 2021). Additional analysis has been focused on the positive impact on the environment of SW associated with the fact that SW reduces the amount of overall regular commuting distances. Given its emphasis on the use of technology, SW offers a prime example of an ecological modernisation policy approach that could help alleviate certain environmental problems (Hynes & Rau, 2014). Nevertheless, with the recent development of notions such as sustainable human resource management and corporate social responsibility, SW has increasingly earned attention as a new human resources approach (Eom et al., 2016). Furthermore, there has been limited attention given to SW in the public sector and particularly on its impact on effectiveness, quality of working life, and family life (Decastri, et al., 2020).

This paper aims to fill the gap in knowledge about SW in public organizations with a specific focus on the experience of the employees of two large Italian research organizations during spring 2020. Although the SW during the pandemic should be considered as an ad hoc emergency institutional response, the perception of employees could help indicate strategies to shape the future implementation of SW. The future possible introduction of formalised SW as the “new normal” requires the preparation of radical organisational changes, including work processes, procedures, and business planning (OECD, 2020a, b)). In the next years public organisations will be required to implement an unprecedented change in workforce management that will need to be grounded as far as possible on research evidence. The previous experience and actual use of SW across countries varied enormously, even for the sectors that are most susceptible to teleworking, and this means that a similar diffusion of SW practices is likely to impact very differently the various European labour markets (Fana et al., 2020).

The paper presents an exploratory case study within one of the countries firstly affected by Covid-19, Italy, where SW was massively adopted in the public sector as a response to the pandemic. The paper presents the result of a survey of employees of the National Research Council (Consiglio Nazionale delle Ricerche – CNR) and the National Institute of Geophysics and Volcanology (Istituto Nazionale di Geofisica e Vulcanologia – INGV), exploring how and to what extent the adoption of SW influenced the division of domestic and family care tasks between men and women during the pandemic emergency.

Through the analysis of primary data, the paper seeks to answer the following research questions: How has SW been experienced by the employees in the difficult setting it has been implemented? What have been the main positive and negative aspects of SW? How does SW impact on the size and quality of their work? And, ultimately, what can be learnt by the experience of SW undergone during the pandemic and how can these lessons be integrated into future SW applications?

The results shed light on how the pandemic affected attitudes of employees in adopting SW and how it influenced the amount and quality of their work, with a special focus on the difficulties they encountered in implementing such a new method of work organization.

## Organizational Specificities and Differences between CNR and INGV

### National Research Council

The National Research Council is the leading public research organisation in Italy, with the responsibility of carrying out, promoting, spreading, transferring, and improving research in the main sectors of knowledge growth and its applications to scientific, technological, economic, and social development of the country. Its activities are divided into macro-areas of interdisciplinary scientific and technological research, ranging from life sciences and information and communication technologies (ICT) to social sciences and humanities. CNR is distributed all over Italy with a network of more than 90 institutes. The workforce is comprised of more than 8,000 employees, of whom more than half (4,794) are researchers and technologists. About 4,000 researchers are engaged in postgraduate studies and research training at CNR, all within the organization’s top-priority areas of interest.

### National Institute of Geophysics and Volcanology

The National Institute of Geophysics and Volcanology’s (INGV) objective is to contribute to the understanding of the dynamics of the earth system and the planet’s solid and fluid physical components, as well as the mitigation of associated natural risks. Differently from CNR, whose research spans all academic disciplines, INGV research focuses on a specific field of knowledge. The activity of the institution can be divided into three broad categories: a) scientific and technological research activities in the fields of seismology, volcanology, and environmental sciences; b) institutional and service research activities for society, public administrations, and industry. The main infrastructures are the National Seismic Network, where the 24-h localization and magnitude assessment service is carried out for seismic events that occur in Italy and surrounding areas; the volcanological and seismological surveillance of Sicilian volcanoes and the surveillance of the Neapolitan volcanoes; c) third mission activities, including training, dissemination, technology transfer, patents, commercial spin-off activities, museum activity and scientific dissemination. These articulated research activities and seismic and volcanological services and monitoring involve almost one thousand individuals (940 people) of whom, as in the case of CNR, more than half (590) are researchers and technologists.

## The SW Model and its Application to the CNR and INGV Before and During the COVID-19 Emergency

### Smart Working in Italy

In recent decades, the world of work has seen new ways of defining and structuring working organization in Italy, and at least three broad categories of new work organization have been implemented: part-time; teleworking; and SW.

Part-time allows the employees to work a lesser number of hours per week compared to full time. Teleworking allows the employees to work remotely from home, even if following the same rules of office working in terms of working hours, and where the employers are responsible for providing workers the necessary workstations. SW begun to be employed more recently in the public sector as well as in the private sector, and consists of an even more flexible work arrangement, where the employees can completely and autonomously manage their time and place of work. As with other work arrangements, SW is formally defined within a hierarchical context within an organization by way of the contract between the employer and the employee.

In this paper, as well as in the survey presented in Sect. 4, according to the laws that regulates its implementation, SW is defined as a model of work in which the employee has the maximum degree of flexibility and of autonomy regarding the spaces and the time in which to carry out their work.

Regulation, in the Italian context, has been one of the most important elements in the creation of current organizational working structures and it guided the implementation of the different types of flexible work arrangements (a full description of the main typologies of flexible work arrangements and their legal bases is provided in the Appendix).

Notwithstanding the number of rules regarding its possible organization and functioning, the use of SW remained modest until the outbreak of the COVID-19 pandemic (Di Mascio et al., 2021). In 2019 SW in Italy was present in 58% of large-sized enterprises, 12% of small and medium-sized enterprises, and 16% of the public sector, comprising a total of 570,000 workers (Smart working Observatory, 2019).

The situation radically changed in March 2020 when, following the COVID-19 emergency and the need to implement social distancing rules, the Government issued the decree of March 1, 2020, de facto establishing, then extending with subsequent decrees, the possibility to apply SW to any subordinate employment relationship, even in the absence of individual agreements.

As of April 29, 2020, according to the Ministry of Labour’s data, a total of 1,827,792 workers were classified in SW, 1,606,617 of which were started following the epidemiological emergency (Ministry of Labour and Social Policies, 2020).

### National Research Council

The first adoption of some forms of SW within the CNR dates to 2010 when the procedural guidelines for the application of teleworking were approved. Its application, however, was quite limited in terms of both scope and size.

The adoption of teleworking, which began in an experimental phase in 2012, required a complicated bureaucratic procedure. The possibility to activate the SW, in fact, was foreseen only in the case of a workers’ mental or physical disability and in cases where continuous care needs of children under the age of 8 and/or family members or cohabitants were required. Moreover, teleworking was available only upon the drafting of specific projects, and the number of possible positions was limited to 2% of the workforce of each CNR Institute.

The cumbersome procedure and the limits imposed by the directive board of directors made the use of teleworking very limited in number. According to a report on “Welfare Services to Increase Wellness in the CNR” drafted by the Performances’ Measurement Office, in 2015 out of about 8,000 employers only 55 had implemented teleworking.

For the period 2019–2020, the number of activated contracts increased from 2 to 10%. Accordingly, the number of employees active in teleworking in 2019 increased to 145, and in March 2020 it rose to 417.

The picture changed with the outbreak of the COVID-19 pandemic when SW became necessary to try to flatten the curve of contagion that from the beginning of March 2020 started to rapidly rise throughout the country. To comply with the Decree promulgated on March 1, 2020, by the President of the Italian Council of Ministers, on March 5, the CNR adopted its own “measures to protect the health of CNR employees and other provisions related to the containment of the coronavirus emergency”. Among the measures introduced, the principal one was the possibility for all CNR employees to activate SW even in the absence of specific contractual agreements.

However, if on the one hand the pandemic forced CNR to widen the employment of SW, on the other hand it did not change the centred management approach. In fact, in this phase the employee was able to choose the place where to carry out his work, but not the time in which they worked, which was fixed by the CNR to be between 8am and 7 pm.

### National Institute of Geophysics and Volcanology

The first adoption of teleworking within INGV dates to 2010, when the institution activated a similar new form of work organization for 10 of the employees. In 2015, INGV decided to extend teleworking to 4 additional employees and in 2017 it included another 10 employees, reaching a total of 24 employees.

The activation of teleworking required complex procedures in which employees were required to compete for the limited positions available. Also in this case, the institution was responsible for the verification of the domestic working environment and to provide the employees with the necessary working tools.

Contrary to CNR, however, in 2018 the INGV started a process that led to the implementation of SW (Di Felice et al., 2018). This path started with a survey of all employees, and interviews with directors, executives, and Board members with the aim of obtaining a widely agreed proposal. The result of this process was the possibility to activate SW for all employees up to 5 days per month.

The situation changed at the beginning of 2020 when the pandemic hit the country, and consequently to the legislation INGV implemented SW for most of its employees. Since then, SW at INGV continues to be the ordinary working condition.

Monthly, the managers of the central departments and the directors’ chamber, after consulting with the managers of the organizational units, identify the work activities that are deemed to be compatible with the SW.

To report on the work performed, the employee submits bimonthly reports on the activities carried out while SW.

## Survey Design and Methods

Data employed in this paper have been collected through the survey "Smart working and gender issues in Italian research institutions during the COVID-19 emergency" (Cellini et al, 2020). The questionnaire had been designed to understand how and to what extent the adoption of SW influences the gender division of domestic and family care tasks among employees of Italian research institutions; and to comprehend, in spite of the peculiar situation in which it has been implemented, how SW was experienced by researchers and how it influenced the size and quality of their work, with a special focus on the difficulties they encountered in implementing such a new method of work organization (the full text of the survey is reported in the Appendix).

To reach these objectives the questionnaire was structured in three macro sections: i) personal information; ii) management of SW activities and household and family workloads; and iii) evaluation of SW activity.

The survey was carried out between April 6 and June 10, 2020, in the middle of the first wave of the COVID-19 pandemic, through a questionnaire administered online via the Lime Survey application. Data collection was carried out by sending a link to the questionnaire. Due to the difficult situation represented by the COVID-19 pandemic, and the fact that the survey was first published during the lockdown implemented in Italy, and because of the desire to quickly follow up on the project, the sample was not selected by employing a probability sample method but rather using the convenience sampling technique. The convenience sample technique is defined as a non-probability sampling method where the sample is taken, rather than randomly selected, from a group of people that are easy to reach and willing to participate to the survey. For what concerns the present study, therefore, the survey was sent to all the workers of the main Italian public research institutions, collecting the responses of those workers that were willing to provide them. This also explains the decision to limit the analysis to CNR and INGV. In fact, while for the two institutions the samples reached was sufficiently large to allow a posteriori evaluation of its statistical representativeness, the same did not apply to the other public research institutions.

The questionnaire was filled in by 2,721 employees of several Italian research institutions. From these observations 2,352 and 225 respectively of the CNR and the INGV were extracted. Compared with their reference universes, the samples represent a significant share of the total workforce: 26.96% for CNR and 24.30% for INGV (Table 1).

**Table 1:** Frequencies and percentages of valid cases by institution, SW survey

Institution	Frequency	Sample %	Employees Number	Employees %
CNR	2,352	91.44	8,600	26.96
INGV	225	8.56	926	24.30

Source: SW survey, CNR Irpps, 2020

To assess if the samples were representative of the respective populations, Z-tests and chi-square tests have been performed on the demographic variables. The test results confirmed that the samples are statistically representative in terms of age and seniority while not representative in terms of gender and professional role.

## Results: Data Processing and Interpretation

The present paper compared the two institutions by analysing the data concerning five different survey items: positive and negative aspects of SW; technological difficulties of SW; SW and perception of work; potentially improvable factors of SW; and workers’ evaluation of the SW experience.

The data shows that most respondents were women, older than 46 years, been working in their institution for more than 11 years and fall under the category of researchers and technologists (Table A2 in the Appendix reports the data on the demographic characteristics in CNR and INGV samples).

### Positive and Negative Aspects of Smart Working as Organizational Response to Pandemic

**Fig. 1:**
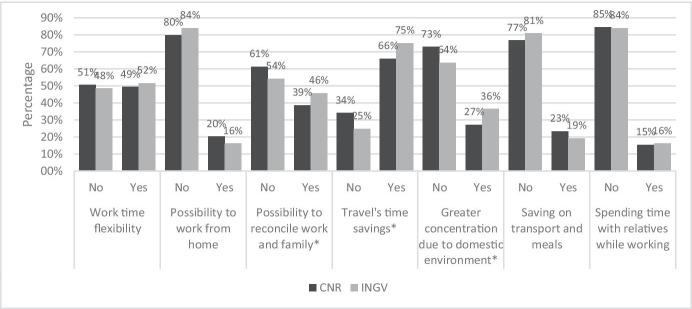
Answer to the question “What are, in your experience, the main positive aspects of smart working?”. Source: SW survey, CNR Irpps, 2020. Note: Asterisks identify those presenting a significant difference at the 95% confidence level

To understand how research institution employees experienced SW during the pandemic, the questionnaire surveyed the main positive and negative aspects they experienced in the implementation of SW. A set of possibly positive and aspects that respondents had to declare if they considered true or not were proposed. Figure 1 shows the comparison of CNR and INGV employees’ on the positive aspects.

The data shows how CNR and INGV employees gave similar answers concerning the different aspects proposed. Most workers considered travel time savings to be a positive aspect of SW while only a relative minority considered the other aspects proposed to be so.

**Fig. 2:**
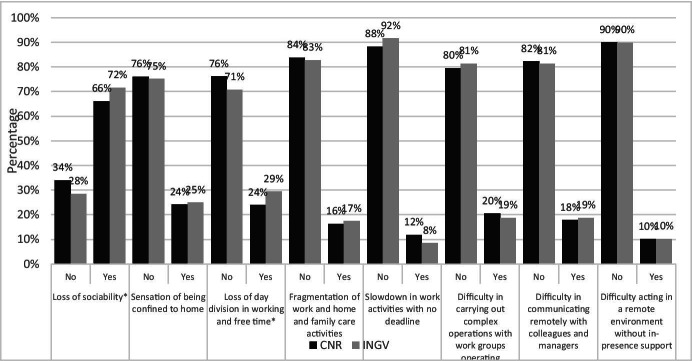
Answer to the question “What are, in your experience, the main negative aspects of smart working?”. Source: SW survey, CNR Irpps, 2020. Note: Asterisks identify those presenting a significant difference at the 95% confidence level

The data also shows some interesting differences between the two institutions. Concerning the possibility of work-life balance, 46% of INGV employees considered it a positive aspect while 39% of CNR’s considered it similarly. Another difference was found regarding travel time savings being considered a positive aspect by 75% and 66% of INGV and CNR employees, respectively. Lastly, 36% of the INGV sample considered the greater concentration offered by the domestic environment a positive aspect as opposed to 27% of the CNR sample. A similar picture appears by looking at Fig. 2, reporting the answers concerning the negative aspects.

Also in this question, CNR and INGV employees gave analogous answers, similarly rating the aspects proposed.

It emerges that most respondents did not consider that most of the aspects proposed were negative aspects of SW. The only factor that was considered negative by most of the respondents, 66% and 72% of CNR and INGV respectively, was the loss of sociability that could not be directly ascribed to SW but rather to the exceptional situation in which it had been implemented. However, even not presenting relevant differences between the two institutions, relevant amounts of respondents within both classified the following as negative aspects: the feeling of being confined at home, the loss of division between working and free time, the fragmentation of work and home and family care activities, the difficulty in carrying out complex operations remotely with working groups, and the difficulty in communicating remotely with colleagues and managers.

The comparison also shows a significant difference in the answers related to loss of social relations, and a disproportional work-life balance. Concerning the first aspect, INGV employees considered it negative in 72% of cases while CNR employees considered it as such in 66% of cases. Even the second aspect was considered negative by a greater share of INGV employees compared to those at CNR, however, in this case the difference accounts only to 5 percentage points.

### Technological Difficulties Linked to Smart Working

Since SW has been massively implemented in the emergency due to the outbreak of the COVID-19 pandemic, without any planning, the questionnaire was also designed to assess if the employees within research institutions experienced technological difficulties in carrying out their working activities. This is an important aspect since the presence of an adequate technological infrastructure is a prerequisite for the implementation of SW itself.

**Fig. 3:**
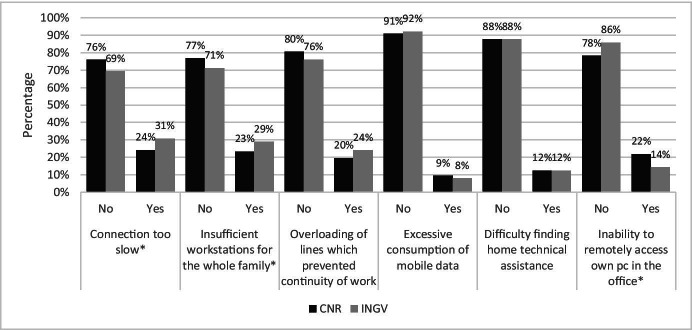
Answer to the question “What do you think were the main technological difficulties of this smart working period?”. Source: SW survey, CNR Irpps, 2020. Note: Asterisks identify those presenting a significant difference at the 95% confidence level

Figure 3 reports respondents’ answers concerning technological difficulties.

The data on different technological aspects proposed show how most of the employees of both institutions did not experience any difficulty. However, roughly a fifth of both samples encountered some difficulties concerning the slowness of home internet connections, the insufficiency of workstations for the whole family, the overloading of lines affecting work continuity, and the inability to remotely access the files stored in the office PCs.

Similarly in this case, when comparing the answers of CNR and INGV respondents some significant differences are identified. Comparatively, INGV more than CNR employees experienced a too slow connection (31% and 24% respectively), insufficiency of workstations for the whole family (29% and 23% respectively), and an overloading of lines that affected work continuity (24% and 20% respectively). On the contrary, CNR more than INGV employees experienced the inability to remotely access their own office PCs, respectively 22% and 14%.

### Smart Working and Perception of Work

Beyond the other aspects, it is important to assess how SW impacted on the amount and quality of the research institutions’ employees’ work. In general terms, an overall evaluation is not an easy task: there are several distinct professional roles with differentiated tasks whose work would need to be evaluated based on different indicators; and the very research work is in itself quite difficult to evaluate since its output considerably varies between disciplines and its evaluation needs to balance qualitative and quantitative indicators that are often difficult to theorize and implement.

Notwithstanding, the evaluation remains fundamental to assess the SW’s effect on work and workers and, in the absence of a direct way to assess this effect in the middle of the pandemic, the questionnaire tried to partially account for it by asking the interviewees to self-evaluate the quality and quantity of their work compared to the pre-SW period.

**Fig. 4:**
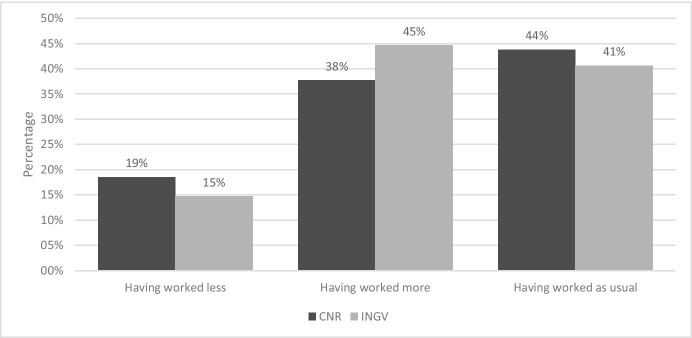
Answer to the question “In the period in which you carried out smart working, did you have the perception of having worked more, less, or as usual?”. Source: SW survey, CNR Irpps, 2020. Note: The test shows a non-significant difference at the 95% confidence level

Figure 4 reports the breakdown of the answers to the question: “In the period in which you carried out smart working, did you have the perception of having worked more, less, or as usual?”.

Data show no significant differences between CNR and INGV, however, comparing the SW and the pre-SW periods, most workers reported to have worked as usual or to have worked more. 38% of the CNR sample and 45% of the INGV sample respectively reported to have worked more, while only 19% of CNR and 25% of INGV samples reported to have worked less.

**Fig. 5:**
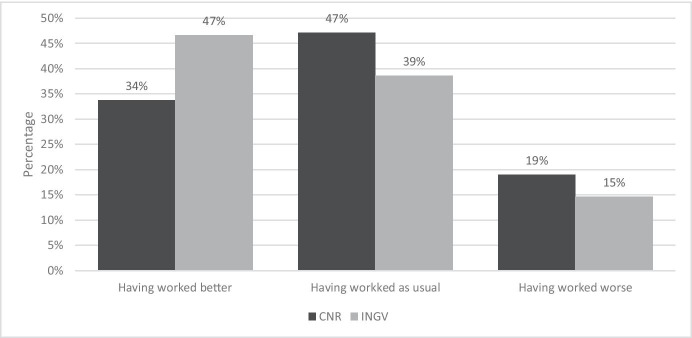
Answer to the question “In the period in which you carried out smart working, did you have the perception of having worked better, as usual, or worse?”. Source: SW survey, CNR Irpps, 2020. Note: The test shows a significant difference at the 95% confidence level

Figure 5 shows the answers to the question: “In the period in which you carried out smart working, did you have the perception of having worked better, as usual, or worse?”.

Most of the respondents of the two institutions reported having worked as usual or having worked better. Bu data in this case reported a significant difference between CNR and INGV samples. 47% of INGV against 34% of CNR workers reported to have worked better, while 19% of CNR against 15% of INGV workers declared to have worked worse. Also, a higher share of CNR against INGV workers declared to have worked as usual, respectively 47% and 39%.

### Potentially Improvable Factors of Smart Working

Even if most respondents perceived to have worked more and better or to have worked as usual in terms of quality and quantity, almost a fifth of them perceived having worked less and worse compared to the pre-SW period.

**Fig. 6:**
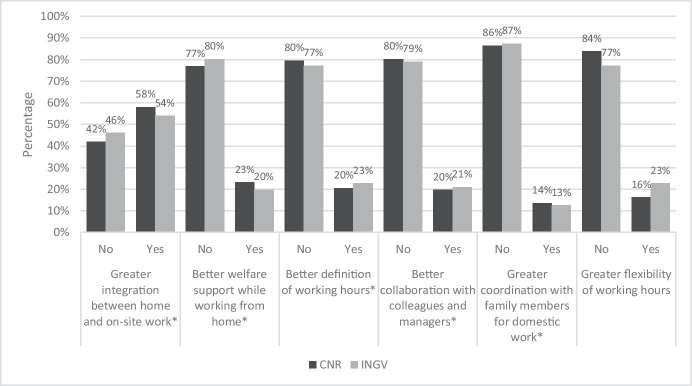
Answers to the question “What do you think are the factors that could be improved in smart working?”. Source: SW survey, CNR Irpps, 2020. Note: Asterisks identify those presenting a significant difference at the 95% confidence level

The survey, therefore, investigated whether, according to the workers, there are specific factors that could be improved in the future implementation of SW. Figure 6 shows their answers.

Most CNR and INGV respondents did not consider the proposed factors to be improvable, except for the integration between home and on-site working. However, even in this case, roughly a fifth of both institutions’ respondents considered to be improvable the following aspects: welfare support in SW, definition of working hours, collaboration with colleagues and managers, and flexibility of working hours.

The comparison of CNR and INGV responses highlighted significant differences with respect to several of the aspects proposed. A higher share of CNR compared to INGV respondents considered the integration between home and on-site working, the welfare support in SW and the coordination with family members for domestic work to be improvable factors. On the contrary, a higher share of INGV compared to CNR respondents considered the definition of working hours to be improvable.

### Evaluation of the Smart Working Experience

Lastly, the survey assessed workers’ overall evaluation of the SW experience, to what extent such evaluation could have been influenced by the measures implemented by the government to face the pandemic, and their propensity to ask for an extension of SW once the pandemic emergency was over.

**Fig. 7:**
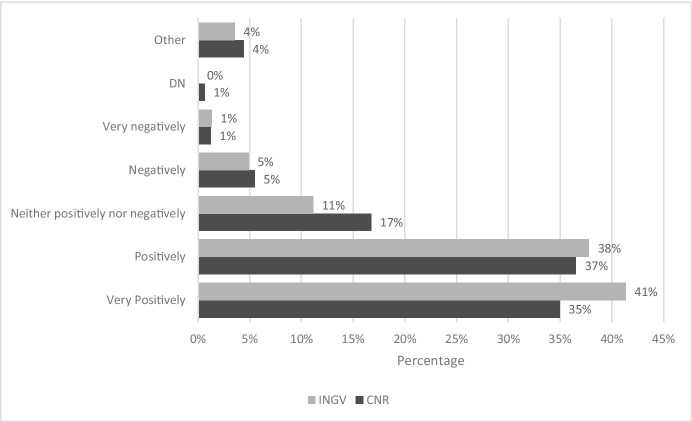
Answer to the question “How do you rate your smart working experience?”. Source: SW survey, CNR Irpps, 2020. Note: The test shows a significant difference at the 95% confidence level

Figure 7 shows the answers to the question: “How do you rate your smart working experience?”.

Concerning the evaluation of the SW experience, Fig. 7 shows how a majority of both CNR and INGV respondents (respectively 72% and 79%) evaluated it as positive or very positive, while only a minority (6%) evaluated it as negative or very negative.

The comparison shows significant differences: INGV employees found the experience to be positive or very positive as a higher share compared to CNR ones. 41% of INGV responses against 35% of CNR responses considered the SW experience to be very positive. A smaller difference in favour of INGV respondents appeared concerning those workers that considered the experience to be positive, but it only accounts to one percentage point. A significant difference was also detected among those workers that considered the SW experience to be neither positive nor negative (17% of CNR against 11% of INGV respondents). No differences instead were reported among the respondents that declared to consider the SW experience negative or very negative, which for both institutions accounted for 6% of the respondents.

**Fig. 8:**
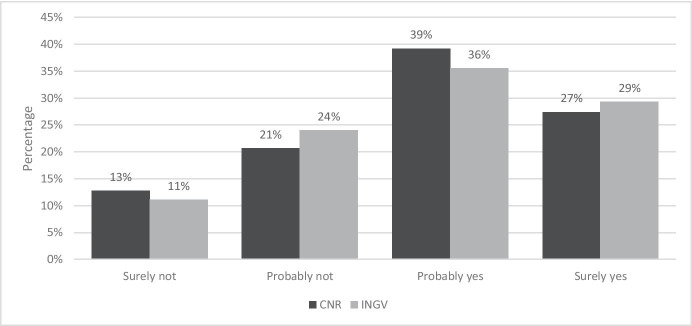
Answer to the question “Do you think having worked in smart working in exceptional conditions may have influenced your perception of smart working?”. Source: SW survey, CNR Irpps, 2020. Note: The test shows a non-significant difference at the 95% confidence level

Figure 8 shows the answers to the question: “Do you think having worked in smart working in exceptional conditions may have influenced your perception of smart working?”.

According to most of the employees within both research institutions (66% for CNR and 65% for INGV) the exceptional conditions in which SW was implemented surely, or probably, influenced the way in which they experienced it. Conversely, unexpectedly, a remarkable share of respondents, 34% of CNR and 35% of INGV, declared that such exceptional conditions did not influence their SW experience. Moreover, the data did not register significant differences between the two research institutions.

**Fig. 9:**
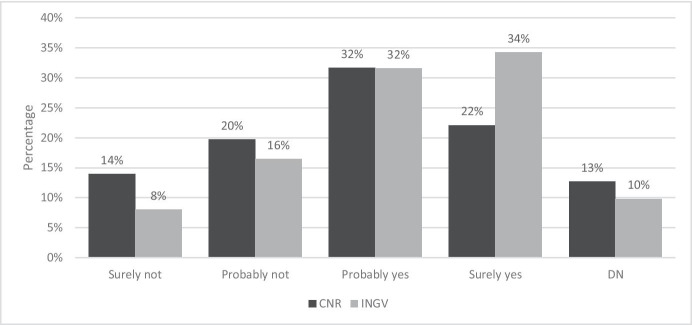
Answer to the question “At the end of this emergency period, do you plan to apply for an extension of smart working?”. Source: SW survey, CNR Irpps, 2020. Note: The test shows a significant difference at the 95% confidence level

Figure 9 shows the answers to the question: “At the end of this emergency period, do you plan to apply for an extension of smart working?”.

More than 70% of CNR and INGV employees considered the SW experience to be positive or very positive. The share of workers that definitely plan to apply for an extension of SW after the end of the pandemic emergency account for only 22% and 34% respectively. Considering together those workers that had already decided and those that are still uncertain, however, the share of respondents increases to 54% for CNR and 66% for INGV.

Even in this case the data shows significant differences between the two research institutions. On the one side, the share of INGV employees that claimed to be convinced to apply for an extension of SW appeared to be higher than that of CNR (34% versus 22%), while on the other side the percentage of CNR workers that declared “probably not” and “definitely not” apply for such an extension are higher than those of INGV, accounting respectively for 20% and 14% against 16% and 8%.

## Discussion and Conclusions

The COVID-19 emergency forced governments to resort to the extensive implementation of SW as an attempt to respond to the spread of the pandemic. This paper focuses on the situation of two Italian public research organizations. At the beginning of March 2020, to tackle the spread of the pandemic, the Italian government, through a series of ad-hoc emergency policies, implemented a total lockdown, obliging private and public bodies to adopt SW for all those workers for which it was feasible.

As a result, the number of SW employees massively increased. It is worthwhile stressing that the obligation to implement SW, imposed by the government, partially distorts the "smart" characteristic of SW, making it a "forced remote work at home", so-called "Covid-work".

The increase in the use of SW promoted a public discussion on SW, involving decision makers, political commentators, mass media and workers, focusing on the functioning of SW, on the opportunity to use it more intensively even after the conclusion of the COVID-19 emergency, and on the evaluation of SW as a new work model.

A discussion on these points, however, requires a better understanding of how SW is perceived and experienced by workers and of its strengths and weaknesses, and of course on how the emergency policies implemented by governments impacted on them. A government’s goal, in fact, must be to develop forms of SW that allow the workers to be more productive; considering and addressing the problems that may arise in the transition from office to SW. In this sense, the widespread implementation of SW offered a natural case-study to observe not only the effects of such measures but also the application of the SW at an unprecedented scale, to learn how to improve on it for future applications.

Through the analysis of primary data collected during the first wave of the pandemic, the paper explored how SW has been experienced by the employees of two Italian research institutions, CNR and INGV, following government implementation of the emergency measures.

In particular, the paper answered the following research questions: how has SW been experienced by the employees in the difficult setting it has been implemented? What has been the main positive and negative aspects they have found in SW? How does SW impact on the quantity and quality of their work? And, finally, what can be learnt by the experience of SW undergone during the pandemic and how can these lessons be integrated into future applications of SW?

In general terms, employees of both institutions mostly appreciated the work time flexibility offered by SW, the travel time savings it allowed and the fact that it permitted a better balancing between work and family time. On the contrary, the only negative aspect observed by most respondents was the loss of sociability. Indeed, this was a direct consequence of government measures implemented to slow down the pandemic curve (i.e., lockdown) rather than a consequence of SW implementation, in fact, during lockdown, unlike in possible previous SW experiences, all social interaction was lost, perhaps making the loss of sociability with colleagues more noticeable. It is interesting to note that such a loss of sociability had been felt to a greater extent by INGV employees. The result is partially puzzling since, due to the same situation of reduced freedom of movement being experienced by all employees, the results were contrary in CNR, SW was employed within INGV even before the pandemic emergency, therefore, one would have expected that INGV employees would have been more accustomed to maintaining social relations remotely.

Concerning the technological difficulties, most respondents within both institutions declared to have not experienced any of the possible issues proposed. However, about a quarter of respondents experienced slow connections, overloading of lines which prevented continuity of work, insufficiency of workstations, and inability to remotely access the files contained in their own PCs in the office. The first two aspects clearly indicate a lack of proper infrastructures within Italy able to support an efficient implementation of SW activities nationwide. This is particularly relevant for those employees who live in small urban or rural areas that are often not covered by fast fibre optic connections nor by fast 4/5 g mobile connections. On the contrary, the insufficient number of workstations and the inability to remotely access the documents stored in own office PCs must be assigned to the unpreparedness of the two institutions. In this respect it is interesting to note how INGV more than CNR employees experienced workstations’ insufficiency while CNR more than INGV employees experienced the inability to remotely access their own documents. Altogether, these results shed light on the urgency to invest in proper infrastructures and tools both on the government and research institutions side.

Despite the negative aspects and the technological difficulties derived from the emergency implementation of SW, the great majority of CNR and INGV employees perceived to have worked more than usual or to have worked as usual, while only a minority perceived to have worked less than usual. Similarly, most researchers perceived to have worked as usual or better than usual, while also only a relative minority perceived to have worked worse than usual. In this case, however, registering a significant difference between CNR and INGV. INGV employees perceived to have worked better in a higher share of cases and to have worked worse in a lower share of cases than CNR employees. These differences are probably partially explained by the fact that, as already pointed out, INGV already had greater experience with SW even before the pandemic, making INGV employees more prepared to operate in such a remote working environment.

To correctly understand the effective influence of governmental measures on the workplace implemented regarding the COVID-19 pandemic, employees self-evaluation cannot be sufficient, and a proper evaluation strategy needs to be developed and implemented. Since the governmental orientation is to increase the use of SW in the public sector even beyond the pandemic emergency, being able to evaluate the output of SW activities will become even more crucial. In this sense, especially for research institutions, the design and implementation of a proper evaluation scheme will be the greater challenge of future SW implementation. The theory of SW, in fact, is based on the pivotal idea that administrative work could and should be evaluated based on clear and measurable objectives rather than on the time the employee spent in his office. Researchers and Technologists already carry out research activities independently and that their results are evaluated within the research quality assessment (VQR) by ANVUR: the evaluation of the research activity in SW by the administrative manager does not appear legally correct and that this cannot be the subject of the performance monitoring and evaluation system (Di Felice et al., 2021).

CNR and INGV workers also highlighted some SW aspects that could and should be improved. Most workers stressed the need for greater integration between home and on-site working, confirming workers’ suffering caused by the loss of sociability expressed when answering about the negative aspects of SW. About a fifth of both institutions’ employees also stressed the need for better forms of welfare support while working from home, a better definition of working hours, a better collaboration with colleagues and managers, a greater coordination with family members for domestic work, and a greater flexibility of working hours.

However, most of both CNR and INGV respondents rated their SW experience as very positive or positive, meaning that in spite of the fact that SW had been suddenly implemented by the government at a high rate, also, in institutions such as CNR that had never implemented SW before the pandemic, research institutions have been able to effectively tackle the situation. It is not surprising that significant differences have been registered between the two institutions considered, and, that the employees of CNR, that had neither SW experience nor regulations before the pandemic, compared to INGV workers, considered their SW experience less positively. Without any significant differences most employees of CNR and INGV declared that their perception of SW has probably or surely been influenced by the exceptional condition in which such experience had been lived.

Lastly, the analysis shows how INGV employees are keener than CNR ones to apply for an extension of SW once the emergency due to the COVID pandemic is over. This difference is probably due to the fact that, in the absence of a previous SW experience and of properly designed policies to favour its implementation, CNR employees are less keen to adopt a new working organization that, as until today, have not been formalized as a perspective for the new “normal times”.

The result of the analysis demonstrates that public workers in Italy responded well to the sudden implementation of SW, but also that adjustments and improvements need to be made by the government, as well as by public bodies, to make SW more functional for workers in the future. In particular, the government must first of all work on a better contractual definition of SW, setting its boundaries and practical application. Also, the government needs to improve all the national technological infrastructures needed to allow employees to carry out their work remotely. At the same time, public bodies will need to better define SW within the borders of each institution, negotiating its application with the employees. Last but not least, each public body will need to adjust their own infrastructures to facilitate the transition from office work to SW. In the path to such a transition a constructive and precise dialogue between government, institutions and workers will be fundamental for designing an organization of work that would be really ‘smart’ and that will improve workers’ productivity but also their work satisfaction.

## Supplementary Information

Below is the link to the electronic supplementary material.

Supplementary file1 (DOCX 50 kb)
